# Ni–Sr/TiZr for H_2_ from methane *via* POM: Sr loading & optimization†

**DOI:** 10.1039/d4ra04781h

**Published:** 2024-08-13

**Authors:** Norah Alwadai, Abdulaziz A. M. Abahussain, Dharmesh M. Vadodariya, Khaled M. Banabdwin, Anis Hamza Fakeeha, Jehad K. Abu-Dahrieh, Naif S. Almuqati, Ahmad M. Alghamdi, Rawesh Kumar, Ahmed S. Al-Fatesh

**Affiliations:** a Department of Physics, College of Science, Princess Nourah bint Abdulrahman University P. O. Box 84428 Riyadh 11671 Saudi Arabia; b Chemical Engineering Department, College of Engineering, King Saud University Riyadh 11421 Saudi Arabia aalfatesh@ksu.edu.sa; c Department of Chemistry, Indus University Ahmedabad Gujarat 382115 India kr.rawesh@gmail.com; d School of Chemistry and Chemical Engineering, Queen's University Belfast Belfast BT95AG UK; e Institute of Refining and Petrochemicals Technologies, King Abdulaziz City for Science and Technology (KACST) P. O. Box 6086 Riyadh 11442 Saudi Arabia; f Chemical Engineering Department, College of Engineering, Imam Mohammad Ibn Saud Islamic University (IMSIU) Riyadh 11432 Saudi Arabia

## Abstract

Achieving remarkable H_2_ yield with significantly high H_2_/CO over Ni-based catalysts through partial oxidation of methane (POM) is a realistic approach to depleting the concentration of CH_4_ and using H_2_ and CO as synthetic feedstock. This study examined Ni catalysts on titania–zirconia for methane conversion *via* POM at 600 °C and atmospheric pressure. The addition of strontium to the catalyst was explored to improve its performance. Catalysts were characterized by X-ray diffraction, Raman-infrared-UV-vis spectroscopy, and Temperature-programmed reduction-desorption techniques (TPR, TPD). 2.5 wt% Sr addition induced the formation of the highest concentration of extreme basic sites. Interestingly, over the unpromoted catalyst, active sites are majorly generated by hardly reducible NiO species whereas upon 2.5 wt% promoted Sr promotional addition, most of active sites are derived by easily reducible NiO species. 45% CH_4_ conversion and 47% H_2_ yield with H_2_/CO = 3.5 were achieved over 2.5 wt% Sr promoted 5Ni/30TiO_2_ + ZrO_2_ catalyst. These results provide insight into the role of basic sites in enhancing activity through switching indirect pathways over direct pathways for POM. Further process optimization was carried out in the range of 10 000–22 000 SV, 0.35–0.75 O_2_/CH_4,_ and 600–800 °C reaction temperature over 5Ni2.5Sr/30TiO_2_ + ZrO_2_ by using central composite design under response surface methodology. The optimum activity as high as ∼88% CH_4_ conversion, 86–87% yield of H_2_, and 2.92H_2_/CO were predicted and experimentally validated at 800 °C reaction temperature, 0.35O_2_/CH_4_ ratio, and 10 000 space velocity.

## Abstract 2

The catalytic performance of strontium promoted 5Ni/30TiO_2_ + ZrO_2_ catalysts are investigated towards partial oxidation of methane, characterized with sophisticated techniques & optimized by central composite design under response surface methodology.

## Graphical abstract

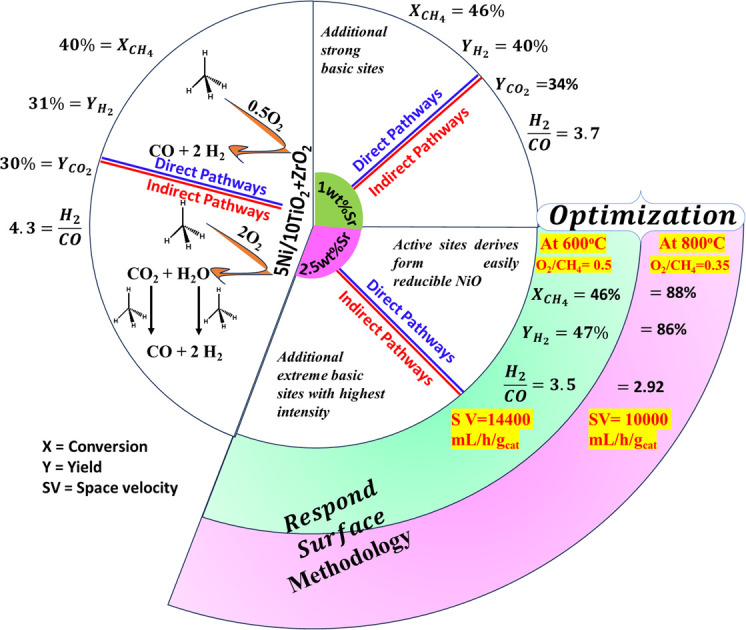

## 1. Introduction

In the current global warming scenario, mitigation of greenhouse gases is urgently needed to save the ecosystem on Earth. Among the greenhouse gases, methane is 25 times more potent than CO_2_.^1^ Catalyst communities across the globe are trying to develop a proper catalytic route for the conversion of CH_4_. The catalytic direct decomposition of CH_4_ into C and H_2_ is struggling due to massive carbon decomposition and quick deactivation. Oxidant-assisted oxidation of CH_4_ also gives way to continuous oxidation of carbon deposits into the syngas (H_2_ and CO). In dry reforming of methane (DRM), CO_2_ is the oxidant; in the steam reforming of methane (SRM), H_2_O is the oxidant; and in partial oxidation of methane, O_2_ is the oxidant for CH_4_ oxidation (eqn (1)–(3)). The typical stoichiometric H_2_/CO ratio in DRM, POM, and SRM is 1, 2, and 3, respectively.

1 CH_4_ + CO_2_ ↔ 2H_2_ + 2CO

2 CH_4_ + 0.5O_2_ ↔ 2H_2_ + CO

3 CH_4_ + H_2_O ↔ 3H_2_ + CO

**Fig. 1:**
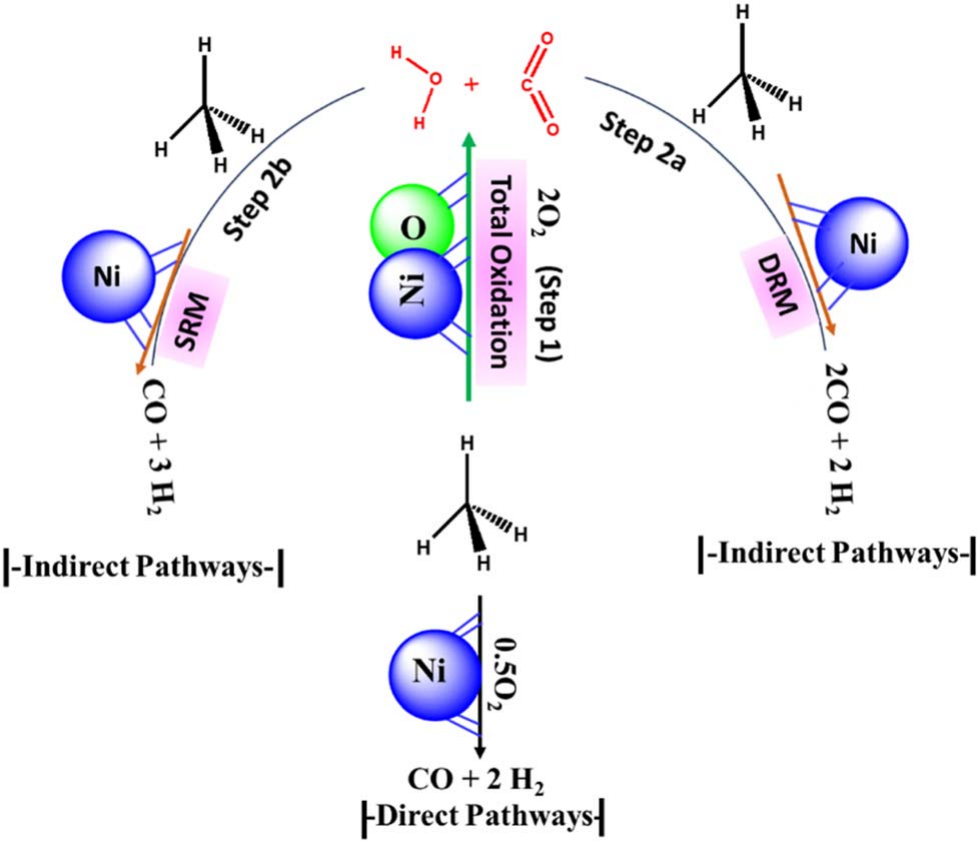
The reaction scheme for POM (A) direct pathways (B) indirect pathways.

According to the stoichiometry of the POM reaction, the H_2_/CO ratio over the POM reaction should be equal to 2. But recently, >3H_2_/CO ratio during POM reaction has drawn marked attention.^2,3^ It indicates the presence of indirect reaction pathways along with the direct POM pathway (Fig. 1) The indirect reaction pathway of POM can be summarized as the total oxidation of CH_4_ into “CO_2_ and H_2_O” (Fig. 1, step 1) followed by the involvement of “CO_2_ and H_2_O” into the oxidation of CH_4_ through DRM (Fig. 1, step 2a) and SRM reaction (Fig. 1, step 2b) respectively. The total oxidation of CH_4_ is catalysed by NiO, whereas POM, DRM, and SRM are catalysed by metallic Ni.^4^ Overall, the direct and indirect pathways of POM decide the final production distribution.

CH_4_ and O_2_ undergo control oxidation over B_2_O_3_-based catalysts and form formate species which is not dissociated further.^5^ Ru dispersed over Al_2_O_3_ (Ru/Al_2_O_3_) carried out the complete oxidation of CH_4_ and O_2_ into syngas. However, adding Re to Ru/Al_2_O_3_ catalyses CH_4_ and O_2_ to control oxidation into formate species, which dissociates into syngas.^6^ Combining noble metals like Rh, Ru, or Pd with a less expensive metal like nickel creates highly effective catalysts for (POM) reactions. Still, the usual high cost of catalysts is a significant limitation in practical applications.^6–8^ Selecting proper supports and promoters for Ni-based catalysts may be as efficient as noble metal-based catalysts. Ni supported over lantana and Ni supported over titania have little life span due to the oxidation of active sites Ni by support lantana or titania.^9–11^ The partial coverage of active sites by diffusion of TiO_2_ and partial phase transformation of titania phase were also reported.^12^ The alumina support may be a suitable carrier for Ni catalysts, but the acidic nature of alumina may be less interactive with acidic CO_2_ gas. So, one route of POM's indirect pathway (step 2a) may be affected. If we select essential support like MgO for the dispersion of Ni, The Ni/MgO catalyst was found to be more prompt to total oxidation of methane than partial oxidation.^9^ Ni has less dispersion over silica than zirconia.^13^ Even ZrO_2_ and yttria-stabilized-ZrO_2_ efficiently carry out POM reactions.^14^ Over yttria-stabilized-ZrO_2_, both CH_4_ and O_2_ undergo control oxidation and form format species, which further break down into syngas. That means it favours the direct methanation route. Again, Ni-supported over ZrO_2_ has a higher H_2_ uptake than Ni-supported over CeO_2_.^10^ The support ZrO_2_ has phase transition issues at high temperatures, and Ni-supported over ZrO_2_ may be seriously affected by metal sintering. Overall, ZrO_2_ and TiO_2_ support have excellent redox properties. Their mobile lattice oxygen is easily accessible in an oxidation reaction, which can minimize the time delay in oxygen transport in POM reaction. However, individually, both have a severe phase transition, and titania has excessive diffusion and active sites' oxidation issues. We have recently developed titania-modified ZrO_2_ support, and the new hybrid support has eliminated all the challenges of phase transition, excessive diffusion, and over-oxidation.^15^ When different types of promoters (Ce, Cs, and Sr) were added, Ni distributed over titania-modified zirconia (Ni/TiZr) continually maintained an H_2_/CO ratio of >3 for 300 minutes on stream.^3^ Sr addition over Ni/TiZr caused the highest activity due to the maximum concentration of active sites and the higher edge of reducibility. Cs addition over Ni/TiZr resulted in inferior catalytic activity due to the lowest density of basic sites. The enhanced reducibility was also observed upon the promotional addition of Sr over Ni/ZrO_2_–Al_2_O_3_ catalyst.^16^ The addition of Sr increases the basicity of the surface, which interacts with more acidic gases like CO_2_ in the indirect POM pathway and helps in the activation of CO_2_. Further, a large size of Sr can stabilize CO_2_ over the catalyst surface as a bidentate carbonate intermediate, as well as enhance the dissociation of C–H by surface oxygen species.^17^ Sr^+2^-mediated CO_2_ was found to be a better oxidizing agent, inhibiting the deposition of carbyne-type carbon.^15^ So, it is necessary to extend the research on the different loadings of Sr promoters over Ni/titania–zirconia catalysts toward POM. After proper catalyst selection, the next step is to set reaction conditions/experimental factors (like temperature, O_2_/CH_4_ ratio, and space velocity) to get maximum H_2_ yield with a high H_2_/CO ratio. Performing fresh reactions after varying reaction conditions in order to optimize activity needs much time, workforce, and expenditure. With the help of statistical tools like response surface methodology (RSM), the optimum activity can be predicted with a very high level of accuracy by few experimental data. The activity is collected as the response of a mathematical equation that considers all the experimental factors and their interactions.^18^ In dry reforming reaction, central composite design and Box–Behnken design under RSM are investigated where predicted values and experiment values are found very close to each other.^19–23^ However, for POM reactions, such statistical investigations are less considered and need to be explored by the catalytic community.

Herein, titania modified-ZrO_2_ is investigated for supporting catalytic active site “5 wt% Ni” and promoter “1–3 wt% Sr” in the target of limiting phase transition of ZrO_2_ and restricting the diffusion for “TiO_2_” and stabilizing the CO_2_ intermediate in favor of POM reaction. The catalysts are characterized by surface area-porosity measurement, X-ray diffraction, Raman, infrared, ultraviolet-visible spectroscopy, H_2_-temperature-programmed reduction, and O_2_-temperature-programmed oxide experiments, and CO_2_-temperature programmed desorption. The thorough characterization and catalytic activity reveal the role of Sr promoter for inducing phase stability and edge and strength of reducibility-basicity of 5Ni/30TiO_2_ + ZrO_2_ catalyst in the favour of POM reaction. The best catalyst is further investigated in process optimization by using a central composite design under response surface methodology in the range of 10 000–22 000 SV, 0.35–0.75 O_2_/CH_4,_ and 600–800 °C reaction temperature.

## 2. Experimental

### 2.1 Materials

Ni(NO_3_)_2_·6H_2_O (purity 98%, Alfa Aesar), Sr(NO_3_)_2_ (Aldrich), 30 wt% TiO_2_–70 wt% ZrO_2_ (Daiichi Kigenso Kagaku Kogyo Co). As per the specification of 30 wt% TiO_2_–70 wt% ZrO_2_ (from Daiichi Kigenso Kagaku Kogyo Co., Ltd), it has a tetragonal-ZrO_2_ phase and diffuse anatase-TiO_2_ phase. The surface area of the 30 wt% TiO_2_–70 wt% ZrO_2_ is 124 m^2^ g^−1^, and 50% of the particles in the catalyst are smaller than 8.6 nm (*D*_50_ = 8.6 nm).

### 2.2 Catalyst preparation

5 wt% equivalent of Ni(NO_3_)_2_·6H_2_O aqueous solution and 1–3 wt% equivalent of Sr(NO_3_)_2_ solution nitrate aqueous solution is added over 30 wt.% TiO_2_–70 wt% ZrO_2_ under stirring at 80 °C temperature. Stirring continues until the solution evaporates and the mixture turns into a paste. The solution was dried at 110 °C for 24 hours and then calcined at 600 °C for three hours. The catalyst is abbreviated as Ni/30TiO_2_ + ZrO_2_ and Ni*x*Sr/30TiO_2_ + ZrO_2_ (*x* = 1, 2, 2.5, 3 wt%).

### 2.3 Catalyst characterization

The BET surface area Powder X-ray diffraction (XRD) analysis of fresh catalyst was conducted by Rigaku (Miniflex) diffractometer using Cu K_α1_ radiation (*λ* = 0.15406 nm) operated at 40 mA and 40 kV. The N_2_ adsorption–desorption and porosity results were obtained using a Micromeritics Tristar II 3020 surface area analyzer. 0.2–0.3 g of catalyst was degassed, and all samples were degassed before analysis using the Barrett, Joyner & Halenda (BJH) method. Temperature-programmed hydrogen reduction (H_2_-TPR) and temperature-programmed carbon dioxide desorption (CO_2_-TPD) measurements were performed on a chemisorption device (Micromeritics AutoChem II) by using a thermal conductivity detector over 70 mg catalyst sample, respectively. In H_2_-TPR, H_2_ absorption is monitored up to 1000 °C under 10% H_2_/He gas, whereas in CO_2_-TPD, CO_2_ desorption is monitored upon raising the temperature to 800 °C under 10% CO_2_/He gas. The transmission electron microscopy was conducted at 200 kV using an aberration-corrected JEM-ARM200F (JEOL) with a CEOS corrector. The spent catalyst sample underwent Raman analysis within the 1250–3000 cm^−1^ range using a Laser Raman Spectrometer (JASCO, Japan) with a 532 nm beam excitation and 1.6 mW laser intensity. The exposure time was set to 10 seconds with 3 accumulations.

### 2.4 Catalyst activity test

The partial oxidation of methane was carried out over a 0.1 g catalyst in a tubular stainless-steel fixed bed reactor (PID Eng. & Tech, 9 mm I.D.). The temperature for the catalytic reaction was provided by the cylindrical furnace circumference of the catalyst bed. The temperature at the catalyst bed was monitored by an axially fixed K-type thermocouple in the catalyst bed. In the target of creating active sites before partial oxidation of methane, a reductive pretreatment of the catalyst was carried out under hydrogen (flow rate 30 mL min^−1^) for 1 h at 800 °C. Further, to remove the hydrogen gas from the catalyst bed, the reactor was purged with N_2_. Then, the temperature of the reactor was stabilized at 600 °C for the POM reaction. The reaction gas mixture which consisted of 50% CH_4_, 25% O_2,_ and 25% N_2_ gases was allowed to pass through the fixed catalyst bed with the total flow maintained at 24 mL min^−1^ and 14 400 mLg^−1^ h^−1^ space velocity. The products were analysed by a gas chromatograph equipped with a propak Q column, molecular sieve columns, and a thermal conductivity detector. The composition of effluent gases was calculated by the normalization method, and the equations for the determination of CH_4_ conversion, CO_2_ conversion, H_2_ yield, CO yield, and H_2_/CO ratio are used as follows:4
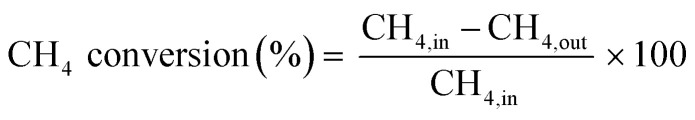
5
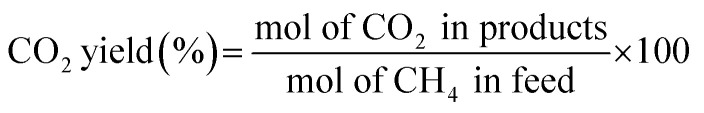
6
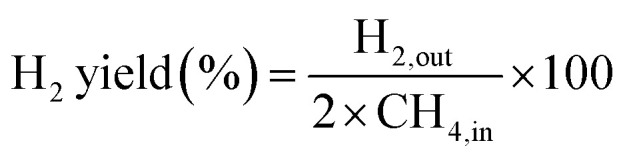
7
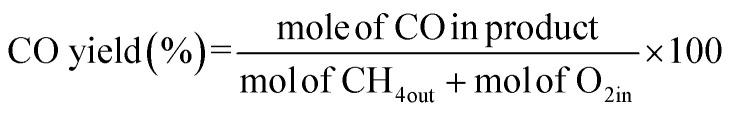
8
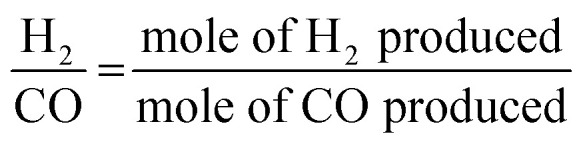


## 3. Result and discussion

### 3.1 N_2_ physisorption

**Fig. 2:**
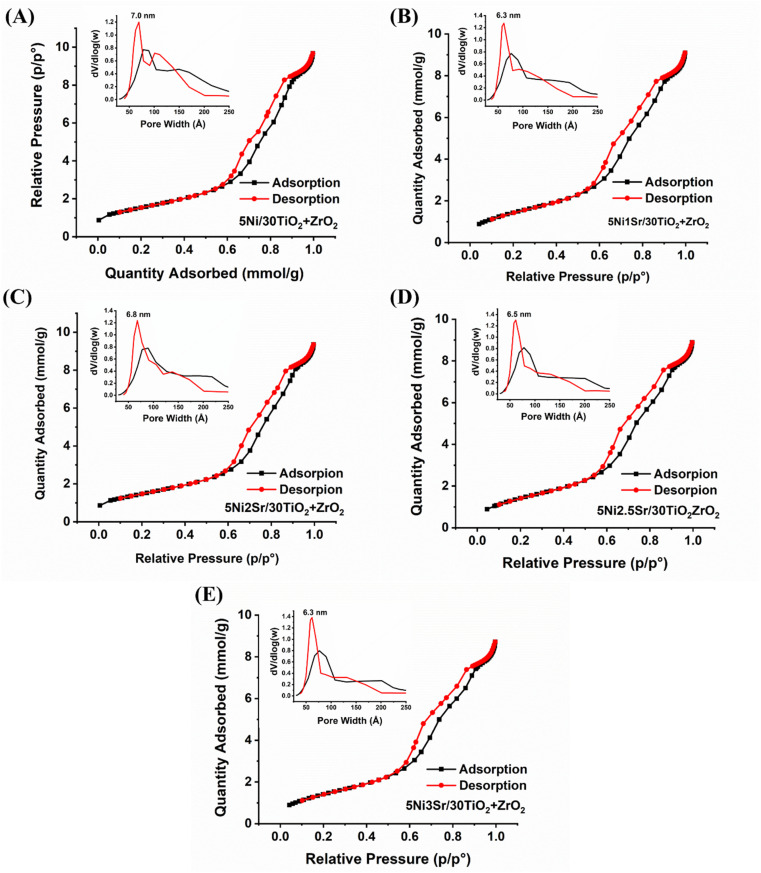
N_2_ adsorption isotherm and porosity distribution (inset figure) of 5Ni/30TiO_2_ + ZrO_2_ and 5Ni*x*Sr/30TiO_2_ + ZrO_2_ (*x* = 1–3 wt%) catalysts.

The N_2_ adsorption isotherm and surface parameters (surface area, pore volume, and pore diameter) of 5Ni/30TiO_2_ + ZrO_2_ and 5Ni*x*Sr/30TiO_2_ + ZrO_2_ (*x* = 1, 2, 2.5, 3) catalysts are shown in Fig. 2 and Table 1. The adsorption–desorption profile of 5Ni/30TiO_2_ + ZrO_2_ and 5Ni*x*Sr/30TiO_2_ + ZrO_2_ (*x* = 1, 2, 2.5, 3) catalysts are characterized by type IV adsorption with H1 hysteresis loop which indicates the presence of mesoporous domains of the cylindrical architect.^24,25^ For such isotherm, a desorption branch is recommended for pore size analysis,^26^ and the “d*V*/dlog(*w*)” *vs.* “*w*” (“*w*” is pore width) plot shows pore size distribution. The 5Ni/30TiO_2_ + ZrO_2_ has a maximum surface area (124 m^2^ g^−1^), pore volume (0.36 cm^3^ g^−1^), and an average 8.6 nm pore diameter. The catalyst has a bimodal distribution of pore size in the range of 7.0 nm and 10.2 nm. Interestingly, upon promotional addition of Sr over 5Ni/30TiO_2_ + ZrO_2_, the pore size distribution becomes narrower and monomodal in the 6.3–6.8 nm range. The pore volume remains almost intact upon the addition of Sr promoters over the 5Ni/30TiO_2_ + ZrO_2_ catalyst. Upon adding 1–2 wt% Sr loading over 5Ni/30TiO_2_ + ZrO_2_, the surface area of the catalyst is decreased marginally. The 5Ni2Sr/30TiO_2_ + ZrO_2_ catalyst has a minimum surface area (119 m^2^ g^−1^) but a maximum pore diameter of 8.6 nm among the promoted samples.

**Table 1:** Surface area, pore volume, and average pore size of 5Ni/30TiO_2_ + ZrO_2_ and 5Ni*x*Sr/30TiO_2_ + ZrO_2_ (*x* = 1–3 wt%) catalysts

Catalyst	Surface area (m^2^ g^−1^)	Pore volume (cm^3^ g^−1^)	Average pore diameter (nm)
5Ni/30TiO_2_ + ZrO_2_	124	0.36	8.6
5Ni1Sr/30TiO_2_ + ZrO_2_	123	0.36	7.8
5Ni2Sr/30TiO_2_ + ZrO_2_	119	0.37	8.6
5Ni2.5Sr/30TiO_2_ + ZrO_2_	122	0.36	7.8
5Ni3Sr/30TiO_2_ + ZrO_2_	121	0.35	7.6

### 3.2 X-ray diffraction

**Fig. 3:**
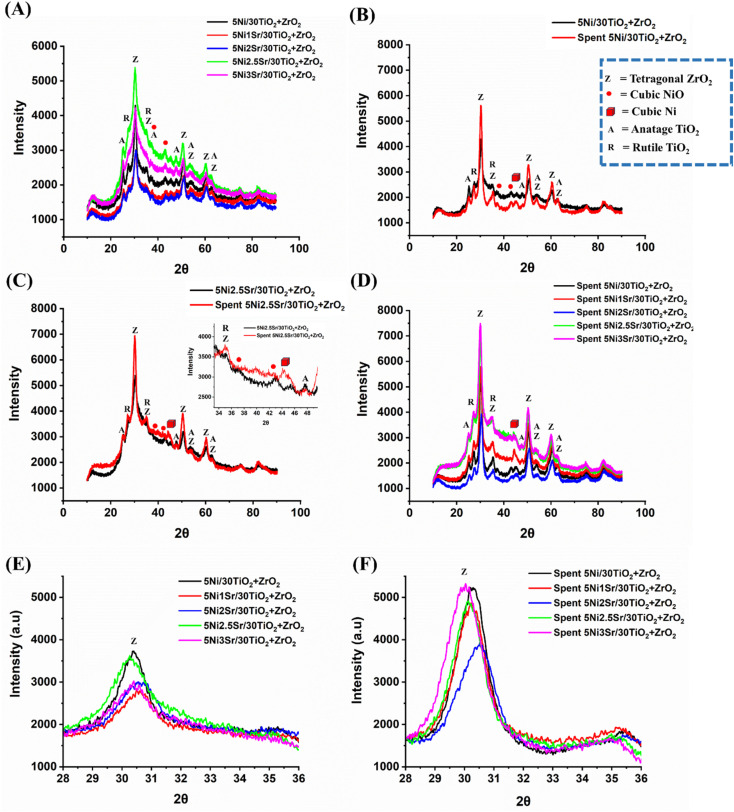
(A) Fresh XRD of all catalysts, (B) fresh-spent XRD of 5Ni/30TiO_2_ + ZrO_2_, (C) fresh-spent XRD of 5Ni2.5Sr/30TiO_2_ + ZrO_2_, (D) spent XRD of all catalysts, (E) fresh XRD peak of ZrO_2_ about 30.5° Bragg's angle (F), spent XRD peak of ZrO_2_ about 30.5° Bragg's angle.

The X-ray diffraction study of fresh and spent 5Ni/30TiO_2_ + ZrO_2_ and 5Ni*x*Sr/30TiO_2_ + ZrO_2_ (*x* = 1–3 wt%) catalysts are shown in Fig. 3. Fresh 5Ni/30TiO_2_ + ZrO_2_ catalyst has intense tetragonal ZrO_2_ phase (at Bragg's angle 2*θ* = 30.28°, 35.15°, 43.05°, 50.50°, 53.60°, 60.32°, 62.76°; JCPDS reference number 01-079-1771) and diffuse peaks for Rutile TiO_2_ phase (at Bragg's angle 2*θ* = 27.51°, 35.15°, 53.60°; JCPDS reference number 00-034-0180), anatase TiO_2_ phase (at Bragg's angle 2*θ* = 25.28°, 47.76°, 53.60°, 63.77°; JCPDS reference number 01-071-1168) and cubic NiO phase (at Bragg's angle 2*θ* = 43.20°; JCPDS reference number 00-047-1049). The crystalline phases for promoter oxides are not observed over Sr-promoted catalysts, which indicates the fine dispersion of promoter oxide. The X-ray diffraction intensity for ZrO_2_ and TiO_2_ phases is found to be a maximum of over 2.5 wt% Sr promoted 5Ni/30TiO_2_ + ZrO_2_ catalyst compared to other catalysts. The loading above 2.5 wt% Sr over 5Ni/30TiO_2_ + ZrO_2_ results in a fall of crystallinity of ZrO_2_ and TiO_2_ phases. Overspent catalysts, the diffraction pattern for metallic cubic Ni (44.68°; JCPDS reference number 00-004-0850) appears, and the peak intensity of tetragonal ZrO_2_, Rutile TiO_2_ and Anatase TiO_2_ phases are intensified. During the POM reaction, the crystalline peak intensity of tetragonal ZrO_2_ is grown slowest over 2 wt% Sr promoted 5Ni/30TiO_2_ + ZrO_2_ than the rest of the catalyst (Fig. 3E and F). It indicates the role of the promoter on support's crystallinity during the POM reaction.

### 3.3 Raman, infrared and ultra-violet spectra

**Fig. 4:**
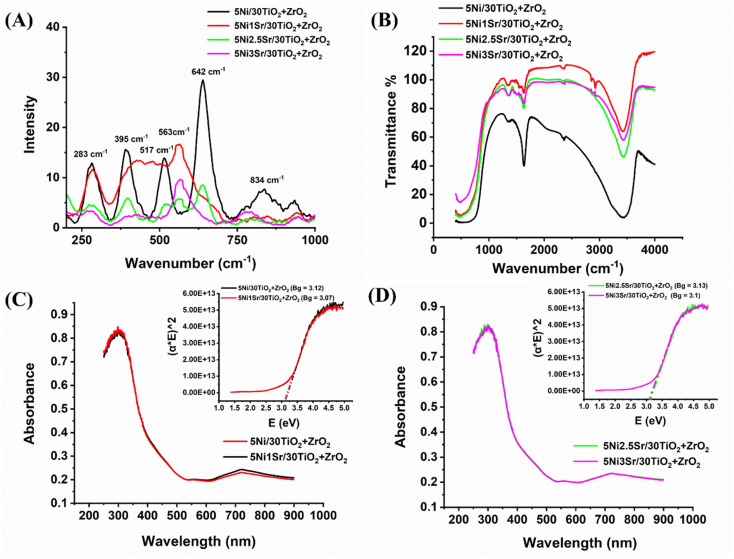
(A) Raman spectra, (B) infrared spectra (full range), (C) ultra-violet spectra of 5Ni/30TiO_2_ + ZrO_2_ and 5Ni1Sr/30TiO_2_ + ZrO_2_ catalysts, (D) ultra-violet spectra of 5Ni2.5Sr/30TiO_2_ + ZrO_2_ and 5Ni3Sr/30TiO_2_ + ZrO_2_ catalysts.

The Raman spectra of 5Ni/30TiO_2_ + ZrO_2_ and 5Ni*x*Sr/30TiO_2_ + ZrO_2_ (*x* = 1, 2, 2.5, 3) catalyst are shown in Fig. 4A. The Raman band at 395 cm^−1^ (B_1g_), 517 cm^−1^ (A_1g_, B_1g_), 642 cm^−1^ (*E*_g_) are observed for anatase TiO_2_ phases whereas the Raman band at 834 cm^−1^ (B_2g_) signifies for rutile phase.^27,28^ The intensity of the anatase phase is higher than the rutile phase over titania–zirconia-supported Ni catalyst. Previously, it was reported that incorporating zirconia atom into titania lattice stabilized the anatase phase.^27,29^ The Raman band at 283 cm^−1^ and 642 cm^−1^ may be associated with partially tetragonal zirconia and partially by segregated TiO_2_ phases, or it may be related to (ZrTi) O_*x*_ material. Sr addition over 5Ni/30TiO_2_ + ZrO_2_ catalyst is found to induce the Raman vibration pattern incredibly. Upon addition of just 1 wt% Sr over 5Ni/30TiO_2_ + ZrO_2_, the *E*_g_ vibration band for anatase TiO_2_ (at 642 cm^−1^) and the B_2g_ vibration band for rutile phase (834 cm^−1^) is suppressed, and the new Raman vibration band about 550 cm^−1^ for amorphous ZrO_2_ phase is appeared.^27^ Upon further loading of Sr up to 3% over 5Ni/30TiO_2_ + ZrO_2_, the intensity of most of the Raman bands declined sharply. Sr addition over 5Ni/30TiO_2_ + ZrO_2_ diminishes the degree of polarizability greatly. Infrared spectra reveal that upon Sr loading, the intensity of the O–H vibration peak (stretching vibration at 2435 cm^−1^ and bending vibration at 1638 cm^−1^) is decreased sharply (Fig. 4B). The depletion of surface hydroxyl's intensity upon Sr loading may be due to the formation of Sr–O–M (M = Ti, Zr, Ni) by condensation of SrOH and MOH (M = Ti, Zr, Ni). Even at simple atmospheric conditions, the unpromoted catalyst (5Ni/30TiO_2_ + ZrO_2_) shows the vibration band bidentate formate at 1355 cm^−1^ whereas Sr promoted 5Ni/30TiO_2_ + ZrO_2_ catalyst has a vibration band for both bidentate formate at 1355 cm^−1^ and ionic carbonate at 1460 cm^−1^ (Fig. 4B).^30,31^ Sr incorporation does not alter the material's UV absorption properties or the energy gap between its valence and conduction bands (Fig. 4C and D). The bandgap in all catalysts remains at about 3.1 eV. That means Sr addition doesn't affect the electronic transition pattern over the catalyst surface.

### 3.4 H_2_-temperature and CO_2_-temperature programmed profiles

**Fig. 5:**
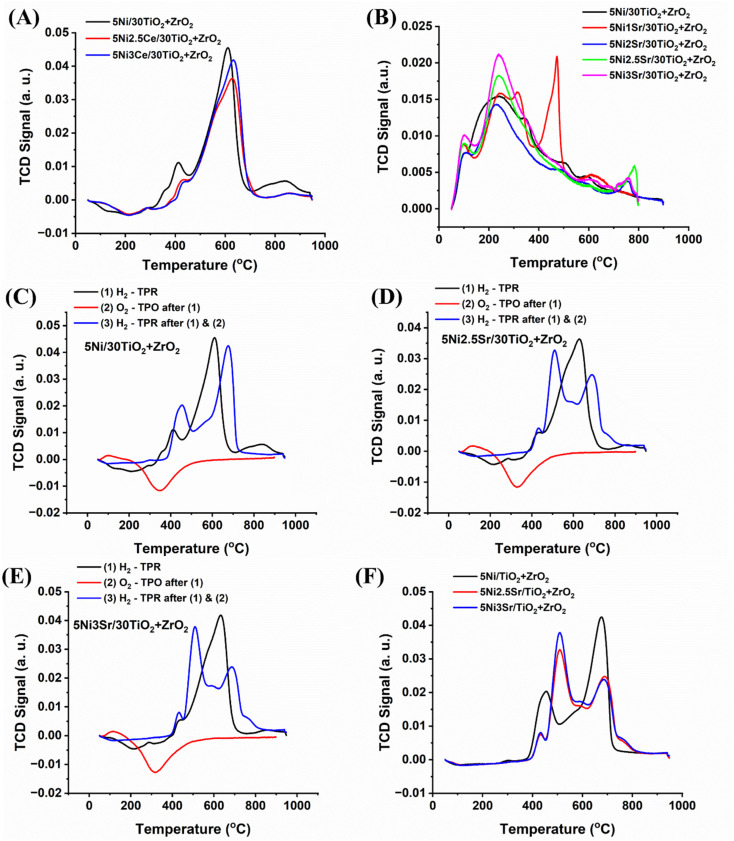
(A) H_2_-temperature programmed reduction profile of 5Ni/30TiO_2_ + ZrO_2_ and 5Ni*x*Sr/30TiO_2_ + ZrO_2_ (*x* = 1, 2, 2.5, 3) catalysts, (B) CO_2_-temperature programmed desorption profile of 5Ni/30TiO_2_ + ZrO_2_ and 5Ni*x*Sr/30TiO_2_ + ZrO_2_ (*x* = 0, 1, 2, 2.5, 3) catalysts. (C) Cyclic H_2_TPR-O_2_TPO-H_2_TPR profile of 5Ni/30TiO_2_ + ZrO_2_, (D) cyclic H_2_TPR-O_2_TPO-H_2_TPR profile of 5Ni2.5Sr/30TiO_2_ + ZrO_2_, (E) cyclic H_2_TPR-O_2_TPO-H_2_TPR profile of 5Ni3Sr/30TiO_2_ + ZrO_2_, (F) the final H_2_TPR (from cyclic H_2_TPR-O_2_TPO-H_2_TPR profile) of 5Ni/30TiO_2_ + ZrO_2_ and 5Ni*x*Sr/30TiO_2_ + ZrO_2_ (*x* = 2.5, 3) catalysts.

The H_2_-temperature programmed reduction study of 5Ni/30TiO_2_ + ZrO_2_ and 5Ni*x*Sr/30TiO_2_ + ZrO_2_ (*x* = 1, 2, 2.5, 3) catalysts are shown in Fig. 5A. The reduction profile of the catalyst at different temperatures indicates the extent of the interaction of reducible species with support. A peak in the reduction process is observed around 600 °C. This suggests the reduction of nickel oxide (NiO) species that moderately interacted with the ESI.† ^32^ The CO_2_-temperature programmed desorption profile of 5Ni/30TiO_2_ + ZrO_2_ and 5Ni*x*Sr/30TiO_2_ + ZrO_2_ (*x* = 1, 2, 2.5, 3) catalysts is shown in Fig. 5B and Table S1.† The CO_2_ desorption profile of the 5Ni/30TiO_2_ + ZrO_2_ catalyst system is populated with weak basic sites cantered at about 100 °C and moderate strength basic sites presented by a broad peak in the range of 150 to 400 °C. The first peak (about 100 °C) is due to the desorption of CO_2_ from surface hydroxyl (constituting weak basic sites), whereas the second peak is due to the desorption of CO_2_ from surface oxide ions (constituting moderate strength basic sites).^33–36^ Upon addition of 1 wt% Sr over 5Ni/30TiO_2_ + ZrO_2_ catalyst, the intensity of moderate strength basic sites increases, and a new desorption peak cantered about 475 °C also appears. It can be termed as a strong basic site constituted by bonded carbonate species (by Sr^+2^) over the catalyst surface.^37^ Overall, it can be said that CO_2_ interaction over the catalyst surface is increased at various basic sites upon the addition of basic Sr oxide. Upon 2 wt% Sr loading over 30TiO_2_ + ZrO_2_, the CO_2_ desorption peak at 420 °C disappeared, and a diffuse peak at about 730 °C appeared, which can be termed as extreme basic sites. 730 °C is reported for the decomposition temperature of SrCO_3_.^38^ Over 5Ni2Sr/30TiO_2_ + ZrO_2_ catalyst, it can also be noticeable that when a new peak of about 730 °C is surged, the intensity of weak basic sites and moderate strength basic sites are not grown. This can be attributed to the engagement of SrO with CO_2_ and forms SrCO_3_, constituting an extreme basic site over the catalyst surface^33^ Further loading of Sr (2.5 wt%), weak basic sites, moderate strength basic sites, and extreme basic sites are grown. With increasing the Sr loading further to 3 wt%, both weak basic sites and moderate strength basic sites are grown, but the intensity of extreme basic sites declined. Overall, the basic site concentration is grown upon Sr loading over and 5Ni/30TiO_2_ + ZrO_2_ catalyst and 5Ni3Sr/30TiO_2_ + ZrO_2_ have the highest amount of basic sites (Table S1†).

POM is carried out over a reduced catalyst system where metallic Ni is the active site. However, it should be noticed that in POM, O_2_ is the oxidizing gas that can oxidize metallic Ni into NiO which turns the active sites inactive. In POM, H_2_ gas is coming out as a reaction product. It is reducing gas also and it can further reduce NiO into Ni. The presence of O_2_ and H_2_ may bring an oxidation–reduction cycle during the POM reaction which may change the reduction profile of the catalyst. To understand the reduction profile and rearrangement of the reduction profile under oxidizing gas (O_2_) and reducing gas (H_2_) during POM, an H_2_TPR-O_2_TPO-H_2_TPR cyclic experiment is carried out.

After sequential reduction–oxidation–reduction treatment (by H_2_TPR-O_2_TPO-H_2_TPR cyclic experiment) of 5Ni/30TiO_2_ + ZrO_2_ catalyst, the reduction peak is shifted to the higher temperature (from 640 °C). More interestingly, a new reduction peak of relatively less intensity appeared at about 400 °C which is attributed to NiO species having weak interaction with the support (Fig. 5C). That means a major part of NiO species undergoes stronger metal–support interaction and some parts of NiO interact with weak interaction. The high-temperature peak (640 °C) is reduced hardly whereas the low-temperature reduction peak (400 °C) is easily reducible. Interestingly if 2.5–3 wt% Sr promoted 5Ni/30TiO_2_ + ZrO_2_ catalysts are sequentially tested for reduction–oxidation–reduction treatment (by H_2_TPR-O_2_TPO-H_2_TPR cyclic experiment), the low-temperature reduction peak is magnified than the high-temperature peak (Fig. 5D–F). It indicates that over 5Ni2.5Sr/10TiO_2_ + ZrO_2_ and 5Ni3Sr/10TiO_2_ + ZrO_2_ catalysts, the amount of easily reducible NiO is more than hardly reducible NiO species. So, 5Ni2.5Sr/10TiO_2_ + ZrO_2_ and 5Ni3Sr/10TiO_2_ + ZrO_2_ catalysts have more active sites which is derived from easily reducible NiO and available from early temperature ranges.

### 3.5 Catalytic activity results

Generally, zirconia has prominent monoclinic phases, which are unstable, and in the same way, titania has both anatase and rutile phases. Interestingly, 30 wt% titania–70 wt% zirconia has only a stable tetragonal zirconia phase and anatase titania phases. That means the presence of titania stabilizes the tetragonal phase of zirconia, whereas the incorporation of zirconia into the titania crystal stabilizes the anatase phase of titania.^27,29^ The stable phases of titania–zirconia make it a good choice for supporting the active site “Ni” for partial methane oxidation.

**Fig. 6:**
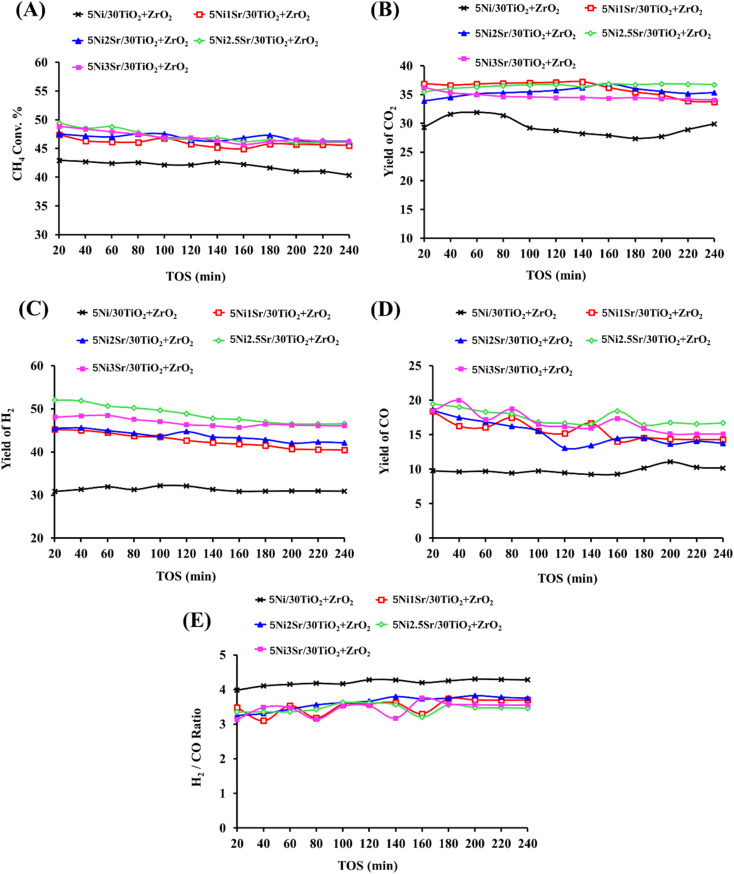
Time on stream of the catalytic activity results of 5Ni/30TiO_2_ + ZrO_2_ and 5Ni*x*Sr/30TiO_2_ + ZrO_2_ (*x* = 1, 2, 2.5, 3) catalysts (A) CH_4_ conversion (%), (B) CO_2_-yield (%), (C) H_2_-yield (%). (D) CO yield (%), (E) H_2_/CO ratio.

The catalytic activity in terms of CH_4_ conversion, H_2_ yield, CO yield and H_2_/CO ratio, and CO_2_ yield over Ni/30TiO_2_ + ZrO_2_ and Ni*x*Sr/30TiO_2_ + ZrO_2_ (*x* = 0, 1, 2, 2.5, 3) catalysts are shown in Fig. 6. NiO is stabilized over a 30TiO_2_ + ZrO_2_ catalyst with moderate strength (as verified by H_2_-TPR). The 5Ni/30TiO_2_ + ZrO_2_ catalyst is reduced before POM in the target of preparing active sites “Ni” for POM reaction. Under the oxidizing gas stream O_2_ (which is one of the feeds of POM) and reducing gas stream H_2_ (which is one of the products of POM), active sites are reorganized and major of the active sites (Ni) is generated by the reduction of hardly reducible NiO. 5Ni/30TiO_2_ + ZrO_2_ catalyst achieves 43–40% CH_4_ conversion during 240 minutes time on stream. In the mean of product distribution, ∼30% H_2_ yield (H_2_/CO ∼4) and ∼30% CO_2_ yield was maintained for up to 240 minutes on stream. From here, two points should be discussed in more depth. The first one is “equal H_2_ yield and CO_2_ yield” over a 5Ni/30TiO_2_ + ZrO_2_ catalyst. It indicates the presence of both partial oxidation and total oxidation of methane over titania–zirconia supported Ni catalyst. Jin *et al.* observed that CH_4_ was catalysed over metallic Ni surface and gave only gaseous H_2_, whereas NiO catalyses CH_4_ and gives CO, CO_2_, and H_2_O.^4^ That means NiO is an active site for total oxidation of methane and oxidation of metallic Ni into NiO (by O_2_) can be easily predicted during POM.^39–41^ Overall, the *in situ* concentration of both Ni and its oxide (NiO) decides the participation of CH_4_ in partial oxidation, in total oxidation, or both. The second point is that the mole of H_2_ is about 4 times the mole of CO over an unpromoted catalyst. If H_2_ and CO come from a POM reaction over a 5Ni/30TiO_2_ + ZrO_2_ catalyst, the stoichiometric ratio of H_2_/CO may not be more than 2. This indicates that H_2_ is also generated by other reaction pathways. This means, the total oxidation reaction takes place and the CO_2_ and H_2_O produced will react with methane in dry reforming and steam reforming, respectively.^42^ These pathways are known as indirect pathways of POM.^43^ Overall, the H_2_/CO ratio ∼4 indicates the increased participation of indirect pathways of POM compared to direct pathways. The water gas shift reaction (CO + H_2_O → CO_2_ + H_2_) is also feasible in this temperature range (600 °C) and it may also increase H_2_/CO ratio.^2,44,45^

Further, in search of optimum product yield, promotional addition of 1–3 wt% Sr is carried out over 5Ni/30TiO_2_ + ZrO_2_ catalyst where Sr is stabilized over catalyst surface by Sr–O–M (M = Ti, Zr, Ni) bond. Adding Sr over 5Ni/30TiO_2_ + ZrO_2_ catalyst brings narrower pore size distribution and enhances the formation of stronger basic sites over the catalyst surface. 1 wt% Sr over 5Ni/30TiO_2_ + ZrO_2_ catalyst may be specified with the presence of strong basic sites (bonded carbonate species by Sr^+2^), whereas 2–3 wt% Sr promoted 5Ni/30TiO_2_ + ZrO_2_ has the presence of extreme basic sites (due to SrCO_3_). 5Ni2.5Sr/30TiO_2_ + ZrO_2_ catalyst has the highest population of extreme basic sites among others. During the POM reaction, the active sites are distributed with different interactions with support in the presence of oxidizing gas (O_2_) and reducing gas (H_2_). H_2_TPR-O_2_-TPO-H_2_TPR cyclic profile shows that upon addition of 2.5 wt% Sr over 5Ni/30TiO_2_ + ZrO_2_, the amount of easily reducible NiO is increased than hardly reducible NiO. So, active sites generated by easily reducible NiO are exposed for POM from a quite early temperature. The CH_4_ conversion is enhanced to 47% and 50% over 5Ni1Sr/30TiO_2_ + ZrO_2_ and 5Ni2.5Sr/30TiO_2_ + ZrO_2_ catalysts, respectively. However, at the end of 100 minutes, CH_4_ conversion over both catalysts reached to 45%. However, at the end of 240 minutes; 5Ni2.5Sr/30TiO_2_ + ZrO_2_ catalyst achieved optimum catalytic activity in mean of higher H_2_ yield (47%) and CO_2_ yield (37%) than 5Ni1Sr/30TiO_2_ + ZrO_2_ catalyst (H_2_ yield = 40%, CO_2_ yield = 34%). This indicates that extreme basic sites over 5Ni2.5Sr/30TiO_2_ + ZrO_2_ catalysts induce POM reactions more towards indirect pathways than 5Ni1Sr/30TiO_2_ + ZrO_2_ catalysts. The H_2_/CO ratio over 5Ni2.5Sr/30TiO_2_ + ZrO_2_ catalyst is equal to 3.5 which indicates the precise presence of indirect pathways of POM. Upon further loading of Sr (3 wt%) over 5Ni/30TiO_2_ + ZrO_2_, the active sites distribution profile is similar than 5Ni2Sr/30TiO_2_ + ZrO_2_. The concentration of extreme basic sites decreases, whereas the concentration of moderate strength basic sites has grown. Overall, the catalytic activity over 5Ni3Sr/30TiO_2_ + ZrO_2_ is found to be relatively inferior to 5Ni2.5Sr/30TiO_2_ + ZrO_2_. 5Ni3Sr/30TiO_2_ + ZrO_2_ showed 46% CH_4_ conversion, 34% CO_2_ yield, 46% H_2_ yield at the end of 240 minutes on stream.

### 3.6 Process optimization

In the current POM experiment, temperature, O_2_/CH_4_ ratio, and space velocity are experimental factors that can be adjusted to get the maximum CH_4_ conversion, H_2_ yield, and H_2_/CO ratio. Now a days, central composite design (CCD) under response surface methodology is utilized frequently in optimization process. It forecasts the optimum response (activity) by using a few experimental data based on variation in experimental factors. Each experimental factor (*x*_*i*_) has a lower limit (*x*_*i*min_) and an upper limit (*x*_*i*max_). (*x*_*i*max_ + *x*_*i*min_)/2  and (*x*_*i*max_ − *x*_*i*min_)/2 are described as centre point of the experiment (*X̄*_o*i*_) and the deviation of each limit from center of experiment (Δ*x*_*i*_) respectively. By using *X̄*_o*i*_ and Δ*x*_*i*_; the original value of experimental factors (*x*_*i*_) are coded into a dimensionless variable (*X*_*i*_) as *X*_*i*_ = (*x*_*i*_ − *X̄*_o*i*_)/Δ*x*_*i*_. Table 2 lists the actual and coded values of the experimental factors.

**Table 2:** The actual and dimensionless variable of the experimental factors

Experimental factors	Actual value (lower limit & upper limit)	Centre point of the experiment (*X̄*_o*i*_)	Deviation from the centre of the experiment (Δ*x*_*i*_)	Dimensionless variable (*X*_*i*_)
Space velocity (ccg^−1^ h^−1^)	10 000	16 000	6000	−1
	22 000			+1
Temperature (°C)	600	700	100	−1
	800			+1
O_2_ : CH_4_	0.35	0.55	0.2	−1
	0.75			+1

RSM predicts the response (*Ŷ*) as per the function of experimental factors modeled under the quadratic polynomial model by using Taylor series expansion as shown below (eqn (9)). The basic terms of statistics related to error metrics (*R*^2^, APE, MAPE, MAE) are briefed in ESI under heading S2.† To refine the model and identify the significant factors & their interaction; analysis of variance (ANOVA) method for various components was carried out and shown in Table S3.† High *F*-values, high *R*^2^ values and low *P*-values indicate that the model terms are significant at approximately the 95% confidence level or at (*p*-values below 0.05). After excluding the insignificant function, models for CH_4_ conversion, yield of H_2_, and H_2_/CO ratio have been proposed using Design-Expert software version 13 (eqn (10)–(12)). Based on these models predicted values of response variables are shown in Table 3. The experimental data and the model's predicted data for CH_4_ conversion, yield of H_2_, and H_2_/CO ratio are shown in Table 3. *R*^2^ values for the expected models of CH_4_ conversion, yield H_2_, and H_2_/CO are 0.9898, 0.9933, and 0.9870, respectively. Table 3 confirms a strong correlation between predicted values and experimental results, with *R*^2^'s near 1. Plotting predicted against actual values is crucial for model assessment, with close alignment to the *X* = *Y* line indicating a good fit. On average, the predicted values have absolute error of 0.95%, 0.81% and 0.78% compared to the actual values. A lower MAPE value indicates a higher level of accuracy in the models.9
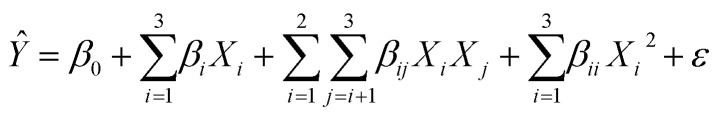
where *X*_1_, *X*_2_, *X*_3_ are the inputs in actual or coded values of the experimental factors, β0 is intercept coefficient, β_*i*,_ i = 1, 2, 3 are the linear coefficients, *β*_*ii*_ are the quadratic coefficients, *β*_*ij*_, *j* = 1, 2, 3 are the interaction coefficients and *ε* is the error term.^18^10
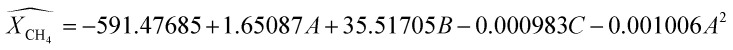
11
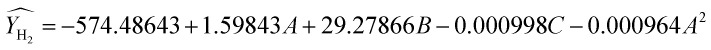
12
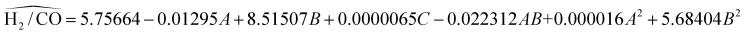
where *A*, *B*, *C* represent temperature, O_2_/CH_4_ ratio and space velocity respectively. 
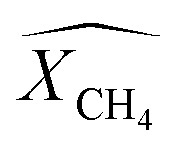
 is conversion of CH_4_ and 
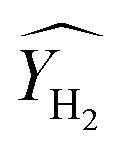
 is yield of hydrogen.

**Table 3:** Experimental and predicted data results for various components of the reaction system (Temp. = temperature, SV = space velocity, Ex. = experimental, Pre. = predicted, Er. = error)

Exp no	Variables			Response								
	*A* (Temp.)	*B* (O_2_/CH_4_)	*C* (SV)	CH_4_ conversion			Yield of H_2_				H_2_/CO	
				Ex.	Pre.	%|Er.|	Ex.	Pre.	%|Er.|	Ex.	Pre.	%|Er.|
1	532	0.55	16 000	5.74	5.98	4.18	3.18	3.08	3.14	3.22	3.28	1.86
2	600	0.35	10 000	39.13	39.62	1.25	35.97	37.68	4.75	2.72	2.69	1.10
3	600	0.75	22 000	40.35	42.03	4.16	35.98	37.41	3.97	3.36	3.32	1.19
4	600	0.35	22 000	27.11	27.82	2.62	24.69	25.7	4.09	2.75	2.76	0.36
5	600	0.75	10 000	55.2	53.83	2.48	51.74	49.39	4.54	3.3	3.24	1.82
6	700	0.21	16 000	61.09	63.11	3.31	59.93	62.07	3.57	3.27	3.24	0.92
7	700	0.55	16 000	78.01	75.19	3.61	75.01	72.02	3.99	2.32	2.29	1.29
8	700	0.55	16 000	77.5	75.19	2.98	75.1	72.02	4.10	2.32	2.29	1.29
9	700	0.89	16 000	90.84	87.26	3.94	81.49	81.98	0.60	2.59	2.66	2.70
10	700	0.55	5909	83.55	85.11	1.87	81.65	82.09	0.54	2.14	2.22	3.74
11	700	0.55	16 000	78.01	75.19	3.61	74.01	72.02	2.69	2.32	2.29	1.29
12	700	0.55	26 091	63.41	65.26	2.92	62.18	61.95	0.37	2.32	2.36	1.72
13	800	0.75	10 000	97.14	99.44	2.37	96.32	99.06	2.84	1.63	1.69	3.68
14	800	0.75	22 000	86.75	90.63	4.47	83.46	87.08	4.34	1.86	1.77	4.84
15	800	0.35	22 000	78.22	76.43	2.29	76.84	75.37	1.91	2.89	3	3.81
16	800	0.35	10 000	87.67	88.23	0.64	85.96	87.35	1.62	2.98	2.92	2.01
17	868	0.55	16 000	91.22	87.63	3.94	89.29	86.53	3.09	2.21	2.18	1.36
				MAPE		2.98			2.95			2.06

#### 3.6.1 Simulation on design expert program

##### 3.6.1.1 One factor effect (2D) plot

**Fig. 7:**
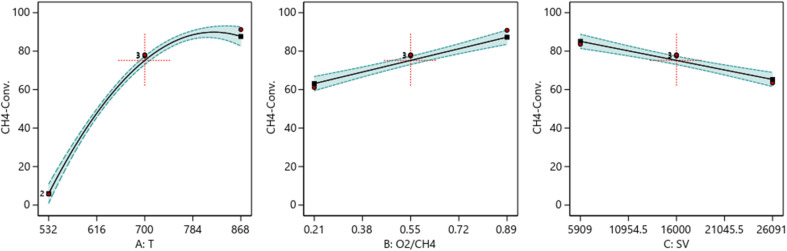
The relationship between the reaction parameters and CH_4_ conversion percentage.

**Fig. 8:**
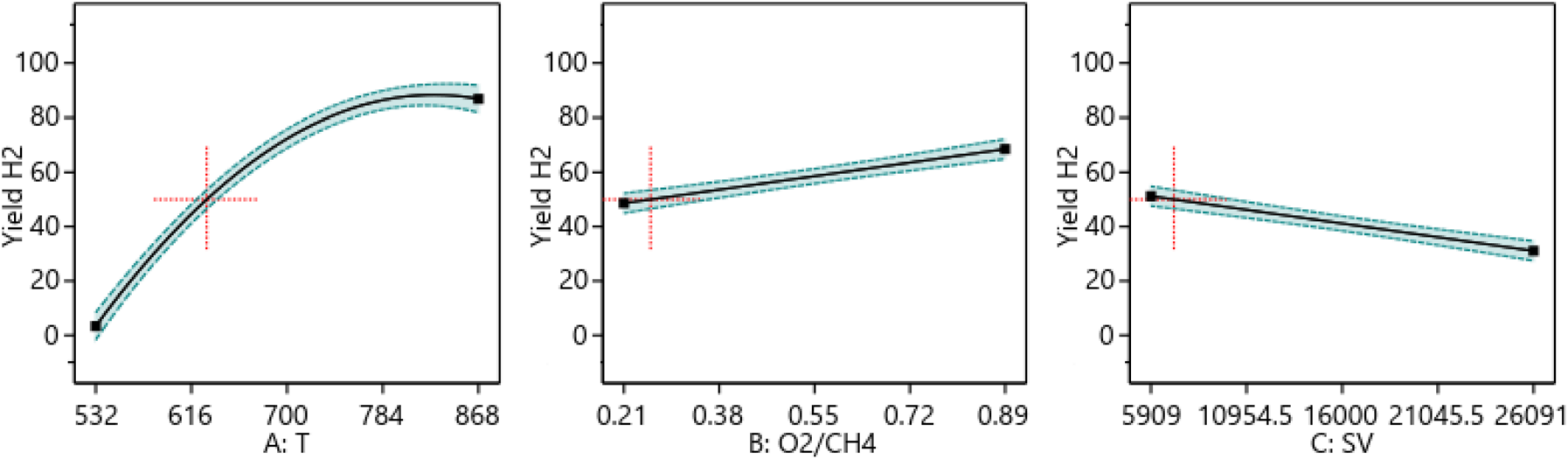
The relationship between the reaction parameters and yield H_2_ percentage.

**Fig. 9:**
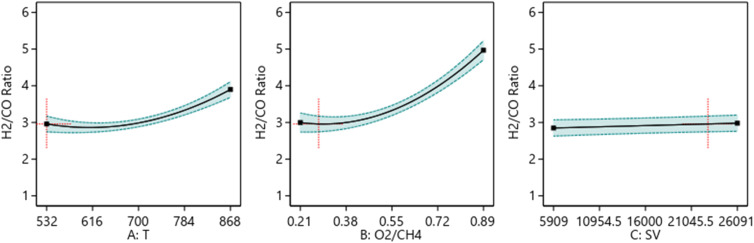
The relationship between the reaction parameters and H_2_/CO ratio.

The effect of each process parameter on the reaction responses is shown in Fig. 7–9. Fig. 7 indicates that increasing temperature, increasing the O_2_/CH_4_ ratio, and decreasing the SV value will increase CH_4_ conversion. Fig. 8 indicates that increasing temperature, increasing the ratio and decreasing the SV value will increase Yield H_2_. Fig. 9 indicates that increasing temperature, increasing the ratio and increasing the SV value will increase H_2_/CO ratio.

##### 3.6.1.2 Two factors effect (3D plot)

**Fig. 10:**
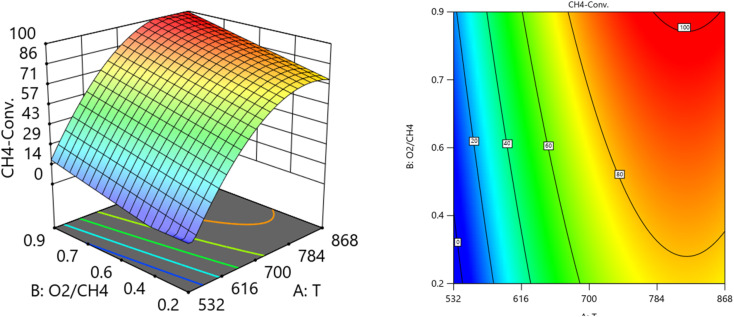
The relationship between the temperature, ratio, and CH_4_ conversion % at SV = 16 201.

**Fig. 11:**
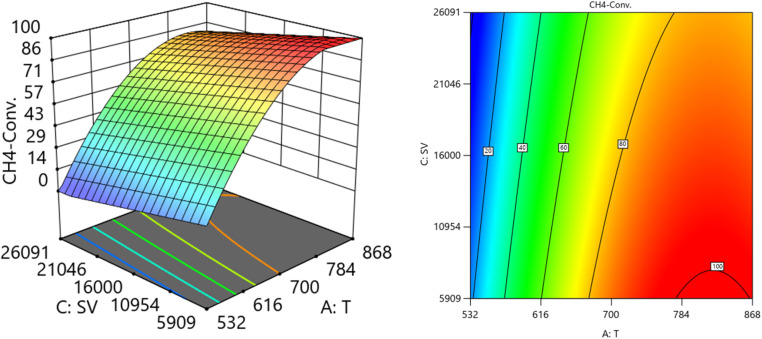
The relationship between the temperature, SV, and CH_4_ conversion % at ratio = 0.6112.

**Fig. 12:**
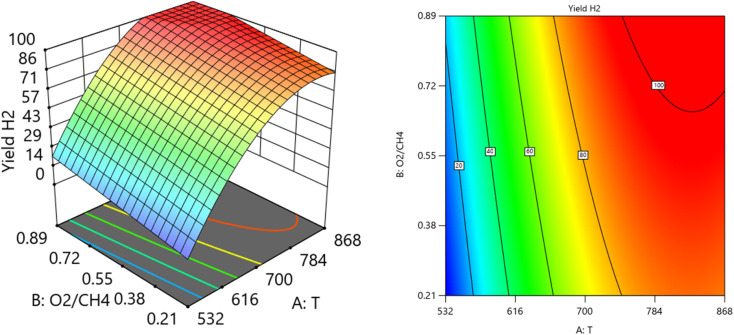
The relationship between the temperature, ratio, and yield H_2_ at SV = 7119.92.

**Fig. 13:**
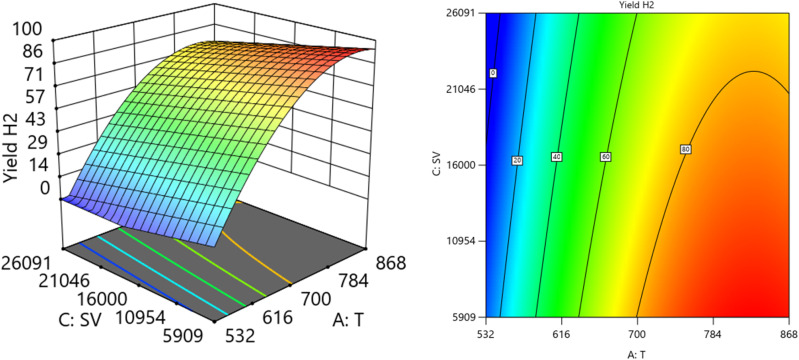
The relationship between the temperature, SV, and yield H_2_ at Ratio = 0.4888.

**Fig. 14:**
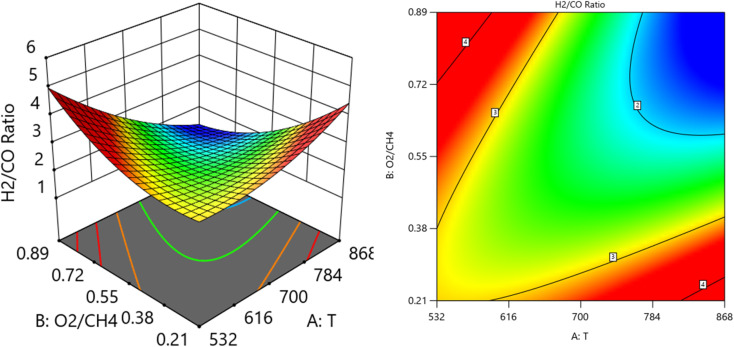
The relationship between the temperature, ratio, and H_2_/CO at SV = 23 000.

**Fig. 15:**
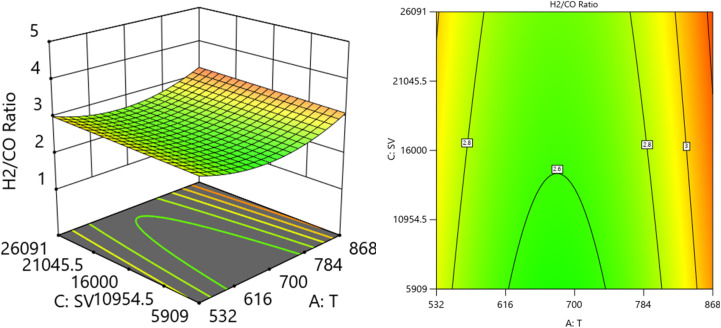
The relationship between the temperature, SV, and H_2_/CO at ratio = 0.3732.

One factor effect (2D) Plot is not suitable for assessing the relative impact of each factor due to coefficient scaling, and the intercept doesn't align with the center of the design space. Through the aid of the resulting equations, and design expert program the response surface plots were constructed for the predicted conversion or formation of the various components comprising the reaction system *versus* two process variables while keeping the third at a constant level or value as shown in the 3D models in Fig. 10–15. Fig. 10–11 show the three-dimensional response surface plots, which represents the effects of the factors (Temperature, SV, and ratio O_2_ : CH_4_) on the variation of CH_4_ conversion. Fig. 10 shows the surface plots which represent the relationship between the response variable (CH_4_ conversion), and the two factors (Temperature and the ratio O_2_ : CH_4_) at SV = 16 201. It is shown with increasing the temperature and increasing the ratio O_2_ : CH_4_, the CH_4_ conversion increase. Fig. 11 shows the surface plots that represent the functional relationship between a designated response variable (CH_4_ conversion), and the two factors variables (Temperature and SV) with O_2_ : CH_4_ fixed at 0.6112. The response surface shows with increasing the temperature and decreasing the SV, the CH_4_ conversion increases. It was observed to increase from 5.74% at 600 °C to 97.14% at 800 °C. All factors have significant effects but the temperature has the major effect on the variation of CH_4_ conversion.

Fig. 12 and 13 show the three-dimensional response surface plots, which represents the effects of the factors (temperature, SV, and ratio O_2_ : CH_4_) on the variation of yield of H_2_. Fig. 12 shows the surface plots which represent the relationship between the response variable (yield H_2_), and the two factors (temperature and the ratio O_2_ : CH_4_) at SV = 7119.92. It is shown that by increasing the temperature and increasing the ratio O_2_ : CH_4_, the Yield H_2_ increases Fig. 13 shows the surface plots that represent the functional relationship between a designated response variable (yield H_2_), and the two factors variables (temperature and SV) with O_2_ : CH_4_ fixed at 0.4888. The response surface shows with increasing the temperature and decreasing the SV, the yield H_2_ increase. It was observed to increase from 3.18% at 600 °C to 96.32% at 800 °C.

Fig. 14 and 15 show the three-dimensional response surface plots, that represents the effects of the factors (temperature, SV, and ratio O_2_ : CH_4_) on the variation of H_2_/CO. Fig. 14 shows the surface plots which represent the relationship between the response variable (H_2_/CO), and the two factors (temperature and ratio O_2_ : CH_4_) at SV = 23 000 at. It is shown the lowest H_2_/CO ratio is found at moderate values of both temperature and O_2_/CH_4_ ratio, which means neither too high nor too low values of both temperature and SV are optimal for minimizing the H_2_/CO ratio. Fig. 15 shows the surface plots that represent the relationship between the response variable H_2_/CO, and the two factors variables (temperature and SV) with O_2_ : CH_4_ fixed at 0.3732. The plot shows that the H_2_/CO ratio is firstly influenced by temperature, with higher temperatures leading to lower H_2_/CO ratios, the space velocity has a lesser effect. It was observed to increase from 1.63% at 600 °C to 3.36% at 800 °C.

##### 3.6.1.3 Model prediction and validation

**Fig. 16:**
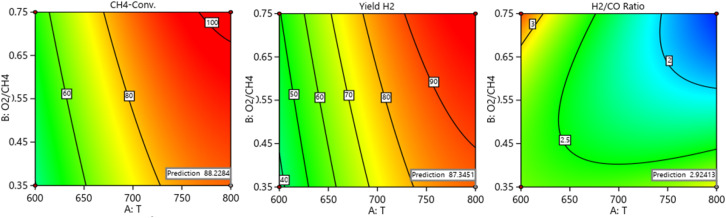
Optimum predicted simultaneous values of CH_4_ conversion, yield H_2_, and H_2_/CO ratio.

Fig. 16 shows optimum predicted simultaneous values of CH_4_ conversion, yield H_2_, and H_2_/CO ratio. Table 4 represents the comparison between predicted with experimental findings. The optimum catalytic activity (88.23% CH_4_ conversion, 87.35% yield H_2_, and 2.92H_2_/CO) towards POM over 5Ni2.5Sr/30TiO_2_ + ZrO_2_ catalyst is predicted at 800 °C reaction temperature, 0.35O_2_/CH_4_ ratio and 10 000 space velocity (SV). At these given reaction conditions, ∼88% CH_4_ conversion, 86% yield of H_2_, and 2.92H_2_/CO are achieved experimentally. The closeness of prediction results and experimental results validate the effectiveness of theoretical models.

**Table 4:** Comparison of theoretical model predictions and experimental findings

		Variables					
Objective function: max (CH_4_-conv.) & max (yield H_2_) & max (H_2_/CO)	Criteria	*T* Value	O_2_ : CH_4_ value	SV value	H_2_/Co max.	Yield H_2_% max.	CH_4_-conv. max.
	Theoretical	800	0.35	10 000	2.924	87.345	88.228
	Experimental	800	0.35	10 000	2.921	86.250	87.850

Further, the best catalyst (5Ni2.5Sr/30TiO_2_ + ZrO_2_) is investigated for long time on stream (28.5 hours) study at 800 °C, 0.35O_2_/CH_4_ ratio, and 10 000 space velocity (Fig. S1A and B†) and the spent catalyst is characterized with thermogravimetry analysis (Fig. S1C†). 44% weight loss is observed over spent 5Ni2.5Sr/30TiO_2_ + ZrO_2_ catalyst after 28.5 h time on stream reaction. But, the CH_4_ conversion and H_2_ yield remains above than 70% during entire time on stream. That means, active sites for POM remains exposed even after high carbon deposit.

## 4. Conclusion

30 wt% titania–70 wt% zirconia (30TiO_2_ + ZrO_2_) support has stable tetragonal ZrO_2_ and anatase TiO_2_ phases, which can stabilize the catalytic active Ni towards POM reaction. During the POM, the active sites undergo re-organization in the presence of oxidizing and reducing gases (O_2_ and H_2_). The majority of these active sites are formed from NiO species that are scarcely reducible. With equal hydrogen (30%) and CO_2_ (30%) yields, the 5Ni/30TiO_2_ + ZrO_2_ catalyst demonstrated 42% CH_4_ conversion, suggesting the existence of both partial (producing H_2_ and CO) and total (producing CO_2_ and H_2_O) oxidation. Due to the presence of a sufficient number of basic sites of moderate strength over 5Ni/30TiO_2_ + ZrO_2_, the total oxidation products (CO_2_ and H_2_O) interact with the surface, subsequently with CH_4_ under indirect pathways and achieve H_2_/CO ∼4. The promotional addition of 1 wt% Sr over 5Ni/30TiO_2_ + ZrO_2_ catalyst enhances additional strong basic sites, whereas 2.5 wt% Sr addition over 5Ni/30TiO_2_ + ZrO_2_ catalyst induces the generation of the highest concentration of extreme basic site. And 5Ni2.5Sr/30TiO_2_ + ZrO_2_ catalyst contains easily reducible NiO too. The easy reducibility promotes methane conversion whereas extreme basicity triggers an indirect pathway of POM by involving total oxidation products, CO_2_ and H_2_O, in the oxidation of CH_4_. The high yield of hydrogen (47%) with a ratio of hydrogen to carbon monoxide (H_2_/CO) of 3.5 suggests that these basic sites promote indirect pathways over direct pathways for methane conversion. Further process optimization is carried out over the best catalyst (5Ni2.5Sr/30TiO_2_ + ZrO_2_ catalyst) in the range of 10 00−22000 SV, 0.35–0.75 O_2_/CH_4,_ and 600–800 °C reaction temperature by using central composite design under response surface methodology. ∼88% CH_4_ conversion, 86–87% yield of H_2_, and 2.92H_2_/CO is predicted and experimentally validated at 800 °C reaction temperature, 0.35O_2_/CH_4_ ratio, and 10 000 space velocity (SV). Hence, the validation experiment confirmed the correctness of the model since the experimental findings obtained under the expected optimal operating circumstances matched the predicted values pretty well.

## Data availability

All data that support the findings of this study are included within the article.

## Conflicts of interest

The authors declare that they have no known competing financial interest or personal relationships that could have appeared to influence the work reported in this paper.

## Supplementary Material

RA-014-D4RA04781H-s001

## References

[cit1] El Hassan N., Kaydouh M. N., Geagea H., El Zein H., Jabbour K., Casale S., El Zakhem H., Massiani P. (2016). Appl. Catal., A.

[cit2] Choudhary V. R., Mondal K. C., V Choudhary T. (2006). Appl. Catal., A.

[cit3] Al-Fatesh A. S., Vadodariya D. M., Banabdwin K. M., Ibrahim A. A., Fakeeha A. H., Adil S. F., Kumar R., Abahussain A. A. M. (2024). Catal. Lett..

[cit4] Jin R., Chen Y., Li W., Cui W., Ji Y., Yu C., Jiang Y. (2000). Appl. Catal., A.

[cit5] Tian J., Tan J., Zhang Z., Han P., Yin M., Wan S., Lin J., Wang S., Wang Y. (2020). Nat. Commun..

[cit6] Li L., MD Dostagir N. H., Shrotri A., Fukuoka A., Kobayashi H. (2021). ACS Catal..

[cit7] Fazlikeshteli S., Vendrell X., Llorca J. (2023). Fuel.

[cit8] Chen Y. G., Tomishige K., Yokoyama K., Fujimoto K. (1997). Appl. Catal., A.

[cit9] Alvarez-Galvan C., Melian M., Ruiz-Matas L., Eslava J. L., Navarro R. M., Ahmadi M., Cuenya B. R., Fierro J. L. G. (2019). Front. Chem..

[cit10] Qian-Gu Y., Chun-Rong L., Wei-Zheng W., Le-Fu Y., Hui-Lin W., Ting-Hua W. (2001). Acta Phys. Chim. Sin..

[cit11] Yang H., An Z., Xu Y., Wu L., Tan L., Tang Y. (2023). Mol. Catal..

[cit12] Shah M., Bordoloi A., Nayak A. K., Mondal P. (2019). Fuel Process. Technol..

[cit13] Jing Q. S., Zheng X. M. (2006). Energy.

[cit14] Zhu J., Van Ommen J. G., Lefferts L. (2004). J. Catal..

[cit15] Elnour A. Y., Fakeeha A. H., Ibrahim A. A., Osman A. I., Abasaeed A. E., Adil S. F., Kumar R., Al-Fatesh A. S. (2024). Res. Chem. Intermed..

[cit16] Al-Fatesh A. S., Ibrahim A. A., Osman A. I., Abasaeed A. E., Alotibi M. F., Alfatesh S. A., Rooney D. W., Fakeeha A. H., Yin C. Y. (2023). Energy Sci. Eng..

[cit17] Al-Fatesh A. S., Alrashed M. M., El-Salamony R. A., Roushdy M. H., Alwan S. M., Osman A. I., Bayazed M., Fakeeha A. H., Ibrahim A. A., Kumar R. (2023). J. CO_2_ Util..

[cit18] V Ayodele B., Khan M. R., Nooruddin S. S., Cheng C. K. (2017). Clean Technol. Environ. Policy.

[cit19] Mohd Jailani M. S. A., Miskan S. N., Bahari M. B., Setiabudi H. D. (2023). Mater. Today Proc..

[cit20] Hossain M. A., Ayodele B. V., Cheng C. K., Khan M. R. (2019). J. Energy Inst..

[cit21] Yusuf M., Farooqi A. S., Alam M. A., Keong L. K., Hellgardt K., Abdullah B. (2022). Int. J. Hydrogen Energy.

[cit22] Hambali H. U., Jalil A. A., Abdulrasheed A. A., Siang T. J., Owgi A. H. K., Aziz F. F. A. (2021). Chem. Eng. Sci..

[cit23] Chong C. C., Cheng Y. W., Setiabudi H. D., Ainirazali N., Vo D.-V. N., Abdullah B. (2020). Int. J. Hydrogen Energy.

[cit24] Mandal S., Sinhamahapatra A., Rakesh B., Kumar R., Panda A., Chowdhury B. (2011). Catal. Commun..

[cit25] Price G. (2006). Catal. Prep..

[cit26] Kumar R. , Surface Characterization Techniques: from Theory to Research, Walter de Gruyter GmbH & Co KG, 2022

[cit27] Kyotani M., Goto H., Suda K., Nagai T., Matsui Y., Akagi K. (2008). J. Nanosci. Nanotechnol..

[cit28] Liu W., Zhong W., Du Y. W. (2008). J. Nanosci. Nanotechnol..

[cit29] Naumenko A., Gnatiuk I., Smirnova N., Eremenko A. (2012). Thin Solid Films.

[cit30] Jia X., Zhang X., Rui N., Hu X., jun Liu C. (2019). Appl. Catal., B.

[cit31] Khatri J., Al-Fatesh A. S., Fakeeha A. H., Ibrahim A. A., Abasaeed A. E., Kasim S. O., Osman A. I., Patel R., Kumar R. (2021). Mol. Catal..

[cit32] Abasaeed A. E., Lanre M. S., Kasim S. O., Ibrahim A. A., Osman A. I., Fakeeha A. H., Alkhalifa A., Arasheed R., Albaqi F., Kumar N. S., Khan W. U., Kumar R., Frusteri F., Al-Fatesh A. S., Bagabas A. A. (2023). Int. J. Hydrogen Energy.

[cit33] Abahussain A. A. M., Al-Fatesh A. S., Rajput Y. B., Osman A. I., Alreshaidan S. B., Ahmed H., Fakeeha A. H., Al-Awadi A. S., El-Salamony R. A., Kumar R. (2024). ACS Omega.

[cit34] Ogo S., Onda A., Iwasa Y., Hara K., Fukuoka A., Yanagisawa K. (2012). J. Catal..

[cit35] Lahuri A. H. , Yarmo M. A. and Tahari M. N. A., Proceedings of the 3rd International Conference on Separation Technology, ed. M. A. A. Zaini, M. Jusoh and N. Othman, Springer Singapore, Singapore, 2021, pp. 175–195

[cit36] Al-Fatesh A. S., Kumar R., Kasim S. O., Ibrahim A. A., Fakeeha A. H., Abasaeed A. E., Atia H., Armbruster U., Kreyenschulte C., Lund H., Bartling S., Ahmed Mohammed Y., Albaqmaa Y. A., Lanre M. S., Chaudhary M. L., Almubaddel F., Chowdhury B. (2022). Ind. Eng. Chem. Res..

[cit37] Miccio F., Murri A. N., Landi E. (2016). Ind. Eng. Chem. Res..

[cit38] Zhu Y., Sunarso J., Zhou W., Shao Z. (2015). Appl. Catal., B.

[cit39] Mrowec S., Grzesik Z. (2004). J. Phys. Chem. Solids.

[cit40] Haugsrud R. (2003). Corros. Sci..

[cit41] Hintz P. A., Ervin K. M. (1995). J. Chem. Phys..

[cit42] Yu S., Hu Y., Cui H., Cheng Z., Zhou Z. (2021). Chem. Eng. Sci..

[cit43] Darkwah W. K., Appiagyei A. B., Puplampu J. B., Otabil Bonsu J. (2023). Langmuir.

[cit44] Patel N., Al-Fatesh A. S., Bamatraf N. A., Osman A. I., Alreshaidan S. B., Fakeeha A. H., Wazeer I., Kumar R. (2024). Catal. Lett..

[cit45] Serhan M. , Sprowls M., Jackemeyer D., Long M., Perez I. D., Maret W., Tao N. and Forzani E., AIChE Annuaul Meettin Conference Proceedings, 2022, vol. 349, p. 118485

