# Supplementary material for: Ni–Sr/TiZr for H2 from methane via POM: Sr loading & optimization
Source: RSC Adv. 2024 Aug 13;14(35):25273–88. doi: 10.1039/d4ra04781h (PMC11320059; doi:10.1039/d4ra04781h)
Supplement: RA-014-D4RA04781H-s001 [file RA-014-D4RA04781H-s001.pdf]

Supplementary Information (SI) for RSC Advances.
This journal is © The Royal Society of Chemistry 2024

Ni-Sr/TiZr for H_2 from Methane via POM: Sr Loading & Optimization

Norah Alwadai[1], Abdulaziz A.M. Abahussain[2], Dharmesh M. Vadodariya[3], Khaled M. Banabdwin[2], Anis Hamza Fakeeha[2], Jehad K. Abu-Dahrieh[4], Naif S. Almuqati[5], Ahmad M. Alghamdi[6], Rawesh Kumar[3*], Ahmed Sadeq Al-Fatesh[2*]

[1]Chemical Engineering Department, College of Engineering, King Saud University, Riyadh 11421, Saudi Arabia
[2]Department of Chemistry, Indus University, Ahmedabad, Gujarat,382115, India

[3]School of Chemistry and Chemical Engineering, Queen's University Belfast, Belfast BT9 5AG, UK

[*]Corresponding Authors: Ahmed S. Al-Fatesh (aalfatesh@ksu.edu.sa), Jehad Abu-Dahrieh (j.abudahrieh@qub.ac.uk) , Rawesh Kumar (kr.rawesh@gmail.com)

Table S1: CO_2 Desorbed Amount calculated form CO_2 – TPD.

Catalyst	CO_2 Desorbed Amount (cm^3/g STP)
$5Ni/30TiO_2+ZrO_2$	0.492
$5Ni1Sr/30TiO_2+ZrO_2$	2.01
$5Ni2Sr/30TiO_2+ZrO_2$	1.47
$5Ni2.5Sr/30TiO_2+ZrO_2$	2.52
$5Ni3Sr/30TiO_2+ZrO_2$	2.74

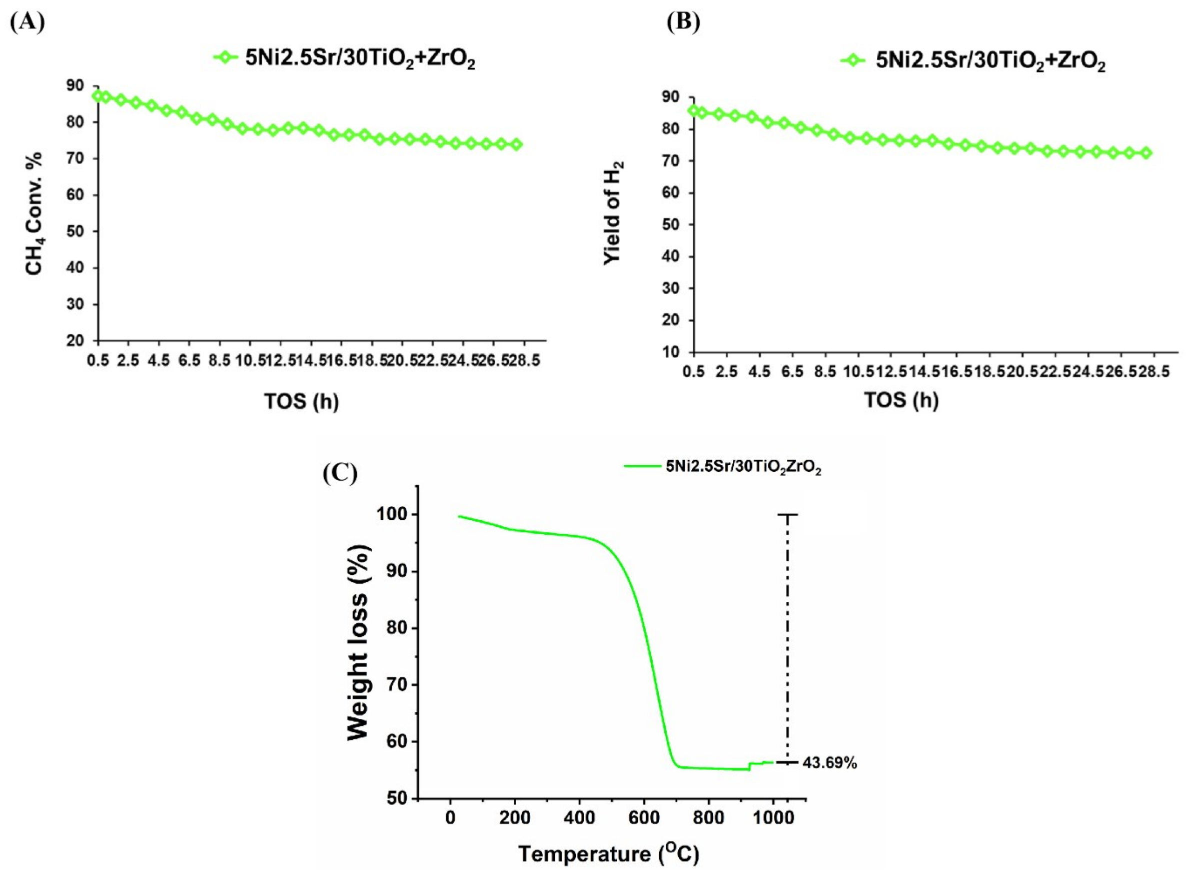

Fig. S1: The long-time on-stream study of Ni52.5Sr/30TiO_2+ZrO_2 (A) CH_4 conversation (%) vs TOS (h) (B) H_2 Yield (%) vs TOS (h). (C) Thermogravimetric analysis of spent 5Ni2.5Sr/30TiO_2ZrO_2 catalyst (after 28.5hour reaction) at 800 °C reaction temperature, 0.35 O_2/CH_4 ratio, and 10000 space velocity
